# Supplementary material for: Dental health care providers’ concerns, perceived impact, and preparedness during the COVID-19 pandemic in Saudi Arabia
Source: PeerJ. 2021 Jun 8;9:e11584. doi: 10.7717/peerj.11584 (PMC8194414; doi:10.7717/peerj.11584)
Supplement: Supplemental Information 3 [file peerj-09-11584-s003.pdf]

Concerns, perceived impact, and preparedness of oral healthcare workers in their working environment during COVID-19 pandemic

Dear Colleagues/Students current study aims to evaluate the oral healthcare workers' concerns, perceived impact, and preparedness in COVID-19 pandemic. Your answers will not be released to anyone but will only be used for the purpose stated above. Your participation in this exercise is voluntary; however, we would be grateful if you participate as we would like to collect as much information as possible.

* Required

1. Category *

Mark only one oval.

- ( ) Clinical Sciences Faculty/Consultants
- ( ) Basic Sciences Faculty
- ( ) Mangerial Clerical staff/Dental Lab technicians/Attendants
- ( ) Dental Hygienists/Dental Assistants
- ( ) General Dental Practitioner

2. Location of Workplace *

Mark only one oval.

- ( ) Riyadh
- ( ) Qassim
- ( ) Jeddah
- ( ) Eastern province
- ( ) Other: ______________________

3. Age *

Mark only one oval.

- ◯ 20-29
- ◯ 30-39
- ◯ 40-49
- ◯ 50-59
- ◯ 60-70

4. Gender *

Mark only one oval.

- ◯ Male
- ◯ Female

5. Marital Status *

Mark only one oval.

- ◯ Single
- ◯ Married
- ◯ Divorced
- ◯ Widowed

6. Place of Work *

Mark only one oval.

- ◯ Government/Public Sector
- ◯ Private Sector

7. Staying with *

Mark only one oval.

- ◯ Family/Friend
- ◯ Alone

8. Work related concerns *

Mark only one oval per row.

	Strongly Agree (6)	Agree (5)	Probably Agree (4)	Probably Disagree (3)	Disagree (2)	Strongly Disagree (1)
My job would put me at great exposure risk	⬭	⬭	⬭	⬭	⬭	⬭
I am afraid of falling ill with Covid-19	⬭	⬭	⬭	⬭	⬭	⬭
I should not be looking after Covid-19 patients	⬭	⬭	⬭	⬭	⬭	⬭
The risk I am exposed to is not acceptable	⬭	⬭	⬭	⬭	⬭	⬭
I accept that risk of contracting Covid-19 is part of job	⬭	⬭	⬭	⬭	⬭	⬭
I might look for another job because of risk	⬭	⬭	⬭	⬭	⬭	⬭
It is acceptable if colleagues resign because of their fear	⬭	⬭	⬭	⬭	⬭	⬭
I am confident employer would look after my needs if I fall ill with COVID-19	⬭	⬭	⬭	⬭	⬭	⬭

9. Non-work concerns *

Mark only one oval per row.

	Strongly Agree (6)	Agree (5)	Probably Agree (4)	Probably Disagree (3)	Disagree (2)	Strongly Disagree (1)
People close to me would be at high risk of getting Covid-19 because of my job	⬭	⬭	⬭	⬭	⬭	⬭
People close to me would be worried for my health	⬭	⬭	⬭	⬭	⬭	⬭
People close to me would be worried as they may get infected by me	⬭	⬭	⬭	⬭	⬭	⬭

10. Non-work concerns- I would be concerned for my *

Mark only one oval per row.

	Strongly Agree (6)	Agree (5)	Probably Agree (4)	Probably Disagree (3)	Disagree (2)	Strongly Disagree (1)
Wife	⬭	⬭	⬭	⬭	⬭	⬭
Parents	⬭	⬭	⬭	⬭	⬭	⬭
Childern	⬭	⬭	⬭	⬭	⬭	⬭
Close Friends	⬭	⬭	⬭	⬭	⬭	⬭
Work colleagues	⬭	⬭	⬭	⬭	⬭	⬭

11. Perceived Impact of COVID-19 *

Mark only one oval per row.

	Strongly Agree (6)	Agree (5)	Probably Agree (4)	Probably Disagree (3)	Disagree (2)	Strongly Disagree (1)
I would be afraid of telling my family about the risk I am exposed						
People would avoid me because of my job						
People would avoid my family members because of my job						
I would avoid telling other people about the nature of my job						
There would be inadequate staff at my workplace to handle the increased demand						
There would be more conflict amongst colleagues at work						
I would feel more stressed at work						
I would have an increase in workload						
I would have to do work not normally done by me						

12. Preparedness for COVID-19 *

Mark only one oval per row.

	Strongly Agree (6)	Agree (5)	Probably Agree (4)	Probably Disagree (3)	Disagree (2)	Strongly Disagree (1)
There is an infection control committee & staff in my clinic						
I have received training for infection control at my clinic						
My clinic has a preparedness plan for a Covid-19 outbreak						
My clinic has informed me of their Covid-19 outbreak preparedness plan						
I am personally prepared for a Covid-19 outbreak						
In the past 6 months I have attended infection control training sessions						
In the past 6 months I have bought disifectants						
In the past 6 months I have bought face masks						
In the past 6 months I have received adequate personal protective equipment training						
I have someone to turn to if unsure of use of personal protective equipment						

This content is neither created nor endorsed by Google.

Google Forms
